# Examining cancer screening disparities by race/ethnicity and insurance groups: A comparison of 2008 and 2018 National Health Interview Survey (NHIS) data in the United States

**DOI:** 10.1371/journal.pone.0290105

**Published:** 2024-02-28

**Authors:** Jingjing Sun, Kevin D. Frick, Hailun Liang, Clifton M. Chow, Sofia Aronowitz, Leiyu Shi

**Affiliations:** 1 Department of Health Policy and Management, Bloomberg School of Public Health, Johns Hopkins University, Baltimore, Maryland, United States of America; 2 Carey Business School, Johns Hopkins University, Baltimore, Maryland, United States of America; 3 School of Administration and Policy, Renmin University of China, Beijing, China; 4 Department of Psychiatry, Cambridge Health Alliance, Cambridge, Massachusetts, United States of America; 5 Department of Psychiatry, Harvard Medical School, Boston, Massachusetts, United States of America; 6 Independent Researcher, Albany, New York, United States of America; The University of Texas MD Anderson Cancer Center, UNITED STATES

## Abstract

**Background:**

Pervasive differences in cancer screening among race/ethnicity and insurance groups presents a challenge to achieving equitable healthcare access and health outcomes. However, the change in the magnitude of cancer screening disparities over time has not been thoroughly examined using recent public health survey data.

**Methods:**

A retrospective cross-sectional analysis of the 2008 and 2018 National Health Interview Survey (NHIS) database focused on breast, cervical, and colorectal cancer screening rates among race/ethnicity and insurance groups. Multivariable logistic regression models were used to assess the relationship between cancer screening rates, race/ethnicity, and insurance coverage, and to quantify the changes in disparities in 2008 and 2018, adjusting for potential confounders.

**Results:**

Colorectal cancer screening rates increased for all groups, but cervical and mammogram rates remained stagnant for specific groups. Non-Hispanic Asians continued to report consistently lower odds of receiving cervical tests (OR: 0.42, 95% CI: 0.32–0.55, p<0.001) and colorectal cancer screening (OR: 0.55, 95% CI: 0.42–0.72, p<0.001) compared to non-Hispanic Whites in 2018, despite significant improvements since 2008. Non-Hispanic Blacks continued to report higher odds of recent cervical cancer screening (OR: 1.98, 95% CI: 1.47–2.68, p<0.001) and mammograms (OR: 1.32, 95% CI: 1.02–1.71, p<0.05) than non-Hispanic Whites in 2018, consistent with higher odds observed in 2008. Hispanic individuals reported improved colorectal cancer screening over time, with no significant difference compared to non-Hispanics Whites in 2018, despite reporting lower odds in 2008. The uninsured status was associated with significantly lower odds of cancer screening than private insurance for all three cancers in 2008 and 2018.

**Conclusion:**

Despite an overall increase in breast and colorectal cancer screening rates between 2008 and 2018, persistent racial/ethnic and insurance disparities exist among race/ethnicity and insurance groups. These findings highlight the importance of addressing underlying factors contributing to disparities among underserved populations and developing corresponding interventions.

## Introduction

Socioeconomic and racial differences in the utilization of healthcare resources, healthcare access, quality of care, and health outcomes in the United States (U.S.) have been well-documented. For many decades, understanding the underlying social determinants of health, eliminating disparities among different socioeconomic groups, and improving overall access to healthcare have been priority goals in the United States [1].

The provision of preventive care services, including cancer screening, is essential because these services provide opportunities for early detection and treatment of health conditions, particularly in vulnerable populations [2, 3]. Many previous studies have shown that substantial racial/ethnic disparities exist in access to care, resource utilization, and health outcomes in the U.S [4–11]. Minority populations experience health disparities in terms of lower cancer screening rates compared with non-Hispanic Whites for many reasons, including lack of health insurance, low income, and low education [11–19]. For example, prior studies have demonstrated that lack of health insurance is associated with a wide range of disparities in the utilization of preventive care services [11, 20, 21], which suggests insurance status could play a mediating role in the relationship between race/ethnicity and cancer screening rates. Nevertheless, despite the plethora of disparities research at various points, little is known about how factors influencing cancer screening have changed in recent years, which can be studied using more recent NHIS databases. There remains a need to evaluate the change in cancer screening rates over time to track the progress during the past decade. This study examines racial/ethnic group and insurance-status differences in preventive screening services in the U.S. population in 2008 and 2018. Other researchers have reported results for racial/ethnic and socioeconomic disparities in cancer screening using NHIS data but have done so for only one year [22–36] or a short period [37]. Some studies analyzed screening rates among two or three major racial/ethnic groups (i.e., Hispanic, non-Hispanic White, non-Hispanic Black) and did not include non-Hispanic Asian [23–27, 30–35, 38–44]. One study has reported patterns and trends in cancer screening using NHIS data from 2000 to 2015 [45], and another examined racial/ethnic disparities in cancer screening in 2000 vs. 2008 [11]. To our knowledge, the present study will be the first to comprehensively examine changes in race/ethnicity and insurance disparities over ten years for breast, cervical, and colorectal cancer screening, based on the 2008 and 2018 NHIS databases. Preventive screening services for breast, cervical, and colorectal cancer will be assessed in accordance with the U.S. Preventive Services Task Force (USPSTF) screening recommendations [46–49]. We hypothesized that racial/ethnic disparities in cancer screening would be attenuated in 2018 compared with 2008 due to significant government-funded programs and policy initiatives aiming at reducing such disparities.

### Objectives

The study’s objective is to assess the relationship between cancer screening rates, race/ethnicity, and insurance coverage and to quantify the changes in screening disparities in 2008 compared with 2018 for breast, cervical, and colorectal cancer using the National Health Interview Survey (NHIS) database.

## Methods

### Data sources

This study is a retrospective cross-sectional analysis of data from the 2008 and 2018 National Health Interview Survey (NHIS) [50, 51]. The NHIS is a cross-sectional household interview survey providing health information on the civilian noninstitutionalized population in the U.S. The National Center for Health Statistics (NCHS) of the Centers for Disease Control and Prevention (CDC) collects the data annually [52]. The employment of a complex sampling design using stratification and clustering ensures a nationally representative sample. The survey consists of a core set of interview questions that gathers baseline demographics, socioeconomic, and health status characteristics for each member of the household. Beginning in the 2019 survey year, the NCHS implemented a redesigned NHIS questionnaire, along with modifications to the weighting and survey design methodology [53]. The comparability of the 2019 NHIS redesign with prior years has not been fully evaluated [54, 55], so therefore, the 2018 NHIS survey was chosen as the reference endpoint for comparing cancer screening rates between a 10 year period (2008 vs. 2018). To the best of our knowledge, there is limited evidence comparing the change in race/ethnicity and insurance disparities in cancer screening utilization in 2008 compared with 2018. In 2008 and 2018, the total unweighted sample sizes were 21,781 and 25,417 adults 18 and older, respectively [50, 51]. Information about cancer screening tests was collected from one randomly selected adult per household, and the final response rate among adults was 62.6% and 53.1% in 2008 and 2018, respectively [51, 56]. All adults who responded to the cancer control supplement of the NHIS and answered questions related to breast, cervical, and colorectal cancer screening will be included in the study. Due to the use of secondary, de-identified, publicly available data for this study, IRB oversight was not required.

### Measures

#### Primary outcomes

The present study will focus on the three screen-detectable cancers: cervical, breast, and colorectal cancer, based on the USPSTF screening guideline recommendations [48–49]. The primary outcome is self-reported preventive care utilization which three cancer screening indicators will measure. During the interview, respondents were asked, “When did you have your most recent [screening test]?” and those who responded affirmatively were also asked the month and the year of the recent screening test. Each outcome will be dichotomized into “recent screening” versus “no recent screening.” Consistent with USPSTF recommended screening intervals in effect at the time of each survey year and previously published literature, the “recent screening” outcomes for cervical, breast, and colorectal cancer will be operationally defined as (1) Pap test in the past three years among women aged 21 to 65 years, or Pap test plus Human Papillomavirus (HPV) test (co-testing) within five years for women aged 30–65 years for a recent screening of cervical cancer [47, 56–58]. (2) mammogram in the past two years among women aged 50 to 74 years for recent screening of breast cancer [45, 48, 55, 59], and (3) colonoscopy in the past ten years, sigmoidoscopy or computed tomography (CT) colonography in the past five years, or home stool-based test (including fecal occult blood tests [FOBT] or fecal immunochemical tests [FIT]) in the past year, or a stool DNA test (sDNA-FIT/Cologuard) in the past three years, among adults aged 50 to 75 years for a recent screening of colorectal cancer [45, 49, 55, 60–62]. The recommended screening intervals can vary depending on the screening modality used. Therefore, “recent screening” for each cancer type is operationally defined as the utilization of at least one screening modality during the USPSTF recommended screening interval.

#### Primary predictors

The main independent variables will be race/ethnicity and types of health insurance coverage. Race/ethnicity will be categorized into Hispanic, Non-Hispanic White, Non-Hispanic Black, and Non-Hispanic Asian, as reported by respondents. Types of health insurance coverage will be categorized into private health insurance, Medicare, Medicaid and other public coverage, and uninsured. Private coverage is defined as any person with a comprehensive private insurance plan (including health maintenance organization, preferred provider organization, and exchange-based coverage). Medicare coverage is defined as anyone who does not have private coverage but only has Medicare coverage, Medicare Advantage coverage, or both Medicare and public coverage (Medicaid and/or other state-sponsored health plans, including State Children’s Health Insurance Program (SCHIP)). Medicaid and other public coverage are defined as any person who has not been classified as having private or Medicare coverage and include persons with only Medicaid coverage, other state-sponsored health plans, or SCHIP, as well as both Medicare and any type of military coverage or Indian Health Service (IHS). Uninsured is defined as persons indicating they are not covered under private, Medicare, Medicaid, SCHIP, state-sponsored health plans, other government programs, or military coverage, as well as persons with only single-service plans (e.g., accident or dental care) or only IHS coverage. Individuals with more than one type of health insurance were assigned to the first appropriate hierarchy as previously described [50, 51, 63].

#### Secondary predictors

Other covariates will include self-reported age, sex, education level, marital status, employment status, family income level, self-reported health status, and English language proficiency. For the cervical test, age will be grouped as 21 to 44 years compared with 45 to 65 years. Mammogram and colorectal cancer screenings will be grouped as 50 to 64 years compared with 65 to 75 years. Education level will be categorized into less than high school diploma, high school diploma or General Educational Development (GED), some college/Associate degree, or Bachelor’s degree or higher. Marital status will be dichotomized into married versus not married. According to the CDC standard for legal marital status, not married includes separated, divorced, single, and widowed. Employment status will be dichotomized into employed versus not employed. Family income will be grouped into $0 - $34,999, $35,000 - $74,999, $75,000 - $99,999, and $100,000 or higher. Health status will be categorized into Excellent/Very good/Good versus Fair/Poor. English language proficiency will be categorized into limited English proficiency versus English proficiency. If the respondent completes the interview in English, he or she will be regarded as English proficient. The respondent will be assigned to limited English proficiency if completed in other languages. The grouping categories chosen are in line with previously conducted research [11, 38, 45, 56].

### Statistical analysis

Our analysis was performed in accordance with the best practices and recommendations for analyzing survey data by NCHS CDC [50, 51, 64, 65]. The statistical analyses were performed using SAS 9.4 (SAS Institute, Cary, NC) and SAS survey procedures, specifically PROC SURVEYMEANS, SURVEYFREQ, SURVEYREG, and SURVEYLOGISTICS. These SAS survey procedures were employed to account for the complex sampling design. Responses initially classified as “Refused,” “Not Ascertained”, or “Do not know” for any cancer screening-related questions were re-coded as a “Missing” value. To account for missing values, the NOMCAR option was used in SAS survey procedures to treat missing values as *not missing completely at random* (NOMCAR). When the NOMCAR option and the DOMAIN statement are specified, the procedure computes variance estimates by analyzing the non-missing values as a domain or subpopulation of interest, accounting for missing and non-missing values in the overall population. As a result, no observations were dropped from the dataset to ensure proper calculation of variance estimates.

An analysis was performed separately for 2008 and 2018, and then the results were compared to identify differences and changes between the two-time points. A bivariate analysis Rao-Scott Chi-square test was performed to compare the distribution of cancer screening rates across race/ethnicity and insurance groups for 2008 and 2018 separately. A similar chi-square test was conducted to compare age-adjusted cancer screening rates in 2008 and 2018 among race/ethnicity and insurance groups [66]. Direct standardization to the U.S. 2000 standard population was used for age adjustment as recommended by The National Center for Health Statistics [67] and in alignment with Healthy People 2020 cancer screening measures [56].

A three-step logistic regression model was conducted to assess the association between recent cancer screening, race/ethnicity, and types of insurance by analyzing 2008 and 2018 NHIS data separately. In Model 1, a simple logistic regression was performed to assess the total impact of race/ethnicity (main independent variable) on cancer screening. In Model 2, logistic regression was performed to assess the combined impact of race/ethnicity and insurance types (two main independent variables) on cancer screening. The model assessed whether accounting for insurance can explain racial/ethnic disparity in cancer screening utilization. Controlling for insurance status in the relationship between race/ethnicity and cancer screening over time enables us to estimate to what extent racial/ethnic disparities in cancer screening change as a response to changes in insurance coverage. For example, some studies suggest Hispanic people had the largest percentage point increase in insurance coverage compared with Blacks, Asians, and Whites between 2008 and 2018 [68], so we may expect racial/ethnic disparities in cancer screening rates to differ in 2018 compared with 2008. Other potential confounders were adjusted in Model 3, including sex, age, education level, marital status, employment status, family income level, self-reported health status, and English language proficiency. Model 3 allowed us to assess the effect of potential confounders on the relationship between race/ethnicity, insurance types, and cancer screening utilization. The change in cancer screening utilization between 2008 and 2018 was assessed to determine whether a statistically significant difference in effect sizes occurred among race/ethnicity and insurance between the two years. Specifically, 2008 and 2018 data were combined for each cancer screening type. Dummy variables for each racial/ethnic group, insurance group, and two years were added as main effects. Each race/ethnicity X year and insurance group X year interaction terms were added to the model. All tests were two-tailed, and p-values less than 0.05 were considered statistically significant.

## Results

### Cancer screening rates in 2008 and 2018

As presented in **Table 1**, cancer screening eligibility remained relatively stable in 2008 and 2018. In 2008, a weighted total of 76,769,989 adults aged 50–75 were eligible for colorectal cancer screening and included in the analysis compared with 95,778,802 in 2018. The weighted number of females aged 21–65 eligible for cervical screening increased from 73,390,857 in 2008 to 81,794,923 in 2018, and the weighted number of females aged 50–74 eligible for mammograms increased from 37,003,082 in 2008 to 46,130,514 in 2018 (Table 1).

**Table 1:** Estimated (weighted) demographic characteristics of study participants by screeningnati type, 2008 and 2018.

Eligible population:	Recent Cervical Screening		Recent Mammogram Screening		Recent Colorectal Screening	
	Female, age 21–65		Female, age 50–75		Male/Female, age 50–75	
	2008	2018	2008	2018	2008	2018
**Weighted sample, n**	73,390,857	81,794,923	37,003,082	46,130,514	76,769,989	95,778,802
**Sex**	*% (SE)*	*% (SE)*	*% (SE)*	*% (SE)*	*% (SE)*	*% (SE)*
Female	100.0 (0.0)	100.0 (0.0)	100.0 (0.0)	100.0 (0.0)	52.1 (0.6)	52.2 (0.6)
**Age group**						
21 to 44 years old	61.7 (0.7)	59.3 (0.7)	-	-	-	-
45 to 65 years old	38.3 (0.7)	40.7 (0.7)	-	-	-	-
50 to 64 years old	-	-	73.5 (0.8)	67.8 (0.7)	71.7 (0.6)	65.7 (0.5)
65 to 75 years old	-	-	26.5 (0.8)	32.2 (0.7)	28.3 (0.6)	34.3 (0.5)
**Race/ethnicity**						
Black, Non-Hispanic	13.4 (0.6)	13.5 (0.6)	11.6 (0.6)	12.1 (0.7)	10.6 (0.4)	11.5 (0.5)
Hispanic	14.8 (0.6)	19.1 (0.9)	8.7 (0.5)	12.4 (0.8)	8.8 (0.4)	11.8 (0.7)
Asian, Non-Hispanic	5.4 (0.3)	7.6 (0.5)	4.1 (0.3)	5.5 (0.5)	3.9 (0.2)	5.3 (0.4)
White, Non-Hispanic	66.4 (0.8)	59.8 (1.0)	75.5 (0.9)	69.9 (1.1)	76.6 (0.6)	71.4 (0.9)
**Insurance status**						
Uninsured	18.2 (0.6)	12.0 (0.6)	9.9 (0.6)	6.3 (0.4)	9.5 (0.4)	6.7 (0.3)
Medicaid & Other public coverage	10.9 (0.4)	15.7 (0.6)	7.2 (0.5)	10.3 (0.6)	8.3 (0.4)	11.1 (0.4)
Medicare	2.5 (0.2)	2.9 (0.2)	14.6 (0.7)	18.2 (0.6)	14.2 (0.4)	18.3 (0.5)
Private Insurance	68.4 (0.7)	69.3 (0.8)	68.3 (1.0)	65.2 (0.9)	68.0 (0.7)	63.9 (0.6)
**Education**						
High school or GED	23.7 (0.6)	20.1 (0.6)	31.1 (0.9)	25.0 (0.8)	29.3 (0.6)	25.5 (0.6)
Bachelor or higher	32.3 (0.8)	40.5 (0.8)	24.1 (0.8)	31.9 (0.9)	27.6 (0.6)	33.4 (0.7)
Some college/Associates	32.7 (0.7)	30.2 (0.7)	29.7 (0.9)	31.4 (0.8)	28.1 (0.6)	29.8 (0.6)
Less than high school	11.3 (0.5)	9.2 (0.5)	15.2 (0.7)	11.8 (0.7)	15.0 (0.5)	11.3 (0.5)
**Marital status**						
Married	57.9 (0.7)	54.1 (0.7)	61.8 (1.0)	61.0 (0.8)	66.4 (0.7)	63.7 (0.6)
Not married	42.1 (0.7)	45.9 (0.7)	38.2 (1.0)	39.0 (0.8)	33.6 (0.7)	36.3 (0.6)
**Employment status**						
Employed	76.0 (0.6)	75.6 (0.7)	56.9 (0.9)	54.5 (0.8)	60.2 (0.7)	58.2 (0.6)
Not Employed	24.0 (0.6)	24.4 (0.7)	43.1 (0.9)	45.5 (0.8)	39.8 (0.7)	41.8 (0.6)
**Family income**						
$0 to $34,999	29.5 (0.7)	23.8 (0.7)	32.6 (0.9)	27.0 (0.8)	31.4 (0.7)	24.8 (0.5)
$35,000-$74,999	34.8 (0.7)	27.8 (0.6)	35.8 (0.9)	28.8 (0.8)	33.7 (0.7)	28.9 (0.6)
$75,000-$99,999	12.7 (0.5)	13.2 (0.5)	11.7 (0.6)	13.8 (0.6)	12.3 (0.5)	14.1 (0.4)
≥$100,000	23.0 (0.7)	35.2 (0.8)	20.0 (0.9)	30.5 (0.9)	22.6 (0.7)	32.2 (0.7)
**Health status**						
Excellent, Very good, or good	89.8 (0.4)	90.4 (0.4)	80.0 (0.8)	82.3 (0.6)	80.3 (0.5)	82.0 (0.4)
Fair or Poor	10.2 (0.4)	9.6 (0.4)	20.0 (0.8)	17.7 (0.6)	19.7 (0.5)	18.0 (0.4)
**Language**						
English proficiency	93.9 (0.4)	93.3 (0.6)	96.2 (0.3)	94.0 (0.6)	96.0 (0.3)	94.6 (0.4)
Limited English proficiency	6.1 (0.4)	6.7 (0.6)	3.8 (0.3)	6.0 (0.6)	4.0 (0.3)	5.4 (0.4)

**Table 2** presents the cancer screening rates by different racial/ethnic and insurance groups for 2008 and 2018. In 2008, the rates of recent cervical tests ranged from 72.2% (non-Hispanic Asians) to 84.6% (non-Hispanic Whites) for race/ethnicity and 63.9% (Uninsured) to 87.7% (Privately insured) for insurance. For recent mammography in 2008, the rates ranged from 61.2% (Hispanics) to 72.8% (non-Hispanic Blacks) for race/ethnicity and 29.0% (Uninsured) to 74.4% (Privately insured) for insurance (Table 2). The rate for colorectal cancer screening was much lower for all racial/ethnicity and insurance groups, ranging from 36.7–56.6% and 18.2–59.7%, respectively. Significant differences across racial/ethnic groups exist for colorectal screenings and cervical tests in 2008 and 2018 (Table 2). Non-Hispanic Whites and non-Hispanic Blacks generally reported the highest rates of recently being screened across all years, followed by Hispanics and non-Hispanic Asians. In 2008, non-Hispanic Blacks had similar rates of recent cervical tests and higher rates of mammograms compared with non-Hispanic Whites, although lower colorectal cancer screening rates. By 2018, non-Hispanic Blacks had comparable rates of recent cervical and mammogram screening, and colorectal screening rates remained lower than non-Hispanic Whites. In 2008, Hispanics had the lowest rates of mammogram and colorectal screenings, although, by 2018, the differences appear to have attenuated and have reached parity with other race/ethnic groups. In 2008 and 2018, non-Hispanic Asians had some of the lowest cervical tests and colorectal screening rates (Table 2). Significant differences were reported across insurance groups for all three cancer types and all years. In 2008 and 2018, privately insured adults consistently reported the highest rates of recent cancer screening for all cancer types, and uninsured adults consistently reported the lowest rates. Medicare beneficiaries, Medicaid and other public coverage-insured adults generally reported rates lower rates than privately insured adults but higher than uninsured adults (Table 2).

**Table 2:** Age-standardized (weighted) cancer screening rates by race/ethnicity and insurance status in 2008 and 2018.

	Recent Cervical Screening		Recent Mammogram Screening		Recent Colorectal Screening	
	2008	2018	2008	2018	2008	2018
	% (SE)	% (SE)	% (SE)	% (SE)	% (SE)	% (SE)
**Overall**	82.9 (0.6)	80.0 (0.7)	69.1 (0.9)	73.0 (0.8)	54.0 (0.7)	64.9 (0.6)
**Race/Ethnicity**	***	***	*		***	***
Black, Non-Hispanic	83.5 (1.7)	82.5 (2.0)	72.8 (2.2)	72.2 (2.2)	50.0 (1.9)	62.0 (1.6)
Hispanic	77.5 (2.0)	77.7 (2.0)	61.2 (3.1)	73.1 (2.4)	36.7 (1.8)	59.5 (1.9)
Asian, Non-Hispanic	72.2 (3.2)	70.3 (2.9)	65.6 (4.6)	67.5 (4.1)	50.6 (3.5)	54.5 (2.9)
White, Non-Hispanic	84.6 (0.7)	81.1 (0.7)	69.6 (1.1)	73.7 (0.9)	56.6 (0.9)	67.0 (0.6)
**Insurance Coverage**	***	***	***	***	***	***
Uninsured	63.9 (2.0)	59.6 (2.3)	29.0 (4.8)	54.0 (6.2)	18.2 (4.2)	34.5 (5.3)
Medicaid & other public	81.5 (2.1)	79.1 (1.8)	68.2 (3.8)	68.9 (3.3)	56.7 (2.5)	64.9 (1.9)
Medicare	74.3 (4.2)	69.6 (4.9)	64.1 (2.5)	69.6 (2.2)	47.3 (1.9)	66.3 (1.6)
Private	87.7 (0.6)	83.8 (0.7)	74.4 (1.1)	78.0 (0.9)	59.7 (0.9)	67.8 (0.8)

*p ≤0.05

**p ≤0.01

***p≤0.001

χ2 test for differences across racial/insurance groups

The weighted estimates are age-standardized to the 2000 U.S. national population.

**SE**: Standard Error

**Fig 1:**
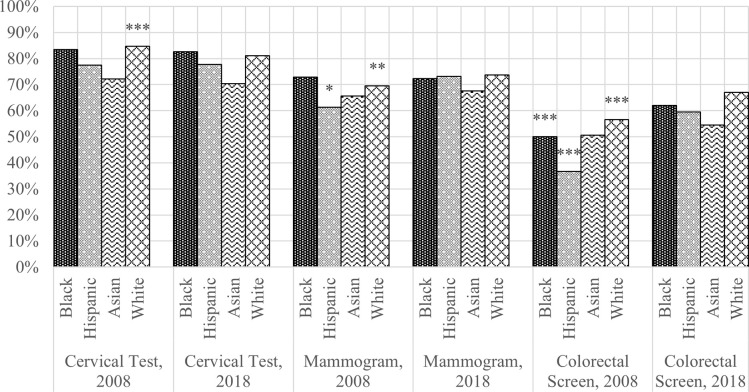
Age-adjusted cancer screening rate by racial/ethnic group, 2008 vs. **2018.** χ2 test for differences between 2008 and 2018 for each racial/ethnic group.

**Fig 2:**
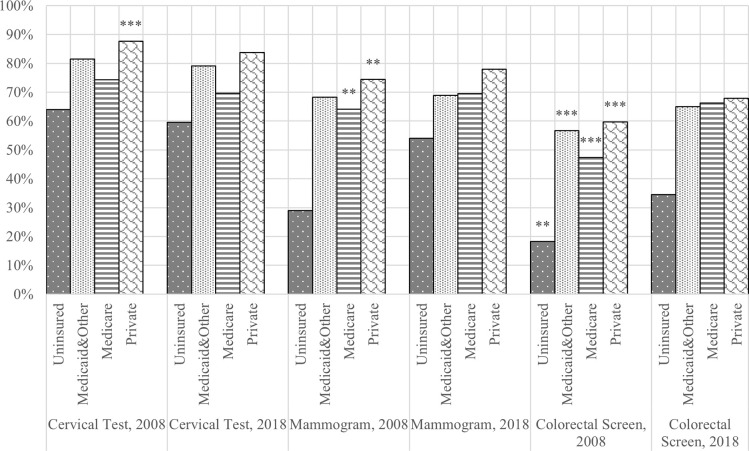
Age-adjusted cancer screening rate by insurance coverage type, 2008 vs. 2018. χ2 test for differences between 2008 and 2018 for each insurance group.

**Figs 1** and **2** report age-adjusted cancer screening rates in 2008 and 2018 and report statistically significant increases or decreases in recent cancer screening rates across all three cancers over time for racial/ethnic groups and insurance groups, respectively. Compared with 2008, rates of cervical tests remained unchanged for non-Hispanic Blacks, Hispanics, and non-Hispanic Asians and decreased by 3.5% for non-Hispanic Whites (Fig 1). Rates of cervical tests consistently decreased for all insurance groups between 2008 and 2018 (Fig 2). Changes in recent mammogram screening rates in Hispanics and non-Hispanic Whites were positive and statistically significant between the two-time points. Across all racial/ethnic groups except non-Hispanic Asians, the rates of colorectal cancer screening significantly increased, ranging from 10.4% for non-Hispanic Whites to 22.8% for Hispanics (Fig 1). Colorectal cancer screening also statistically significantly increased between 2008 and 2018 among all insurance groups, ranging from 8.1% for privately insured adults to 19.0% for Medicare adults (Fig 2).

Over the ten years between NHIS datasets, racial/ethnic disparities persisted in cervical and colorectal tests, and insurance disparities persisted in all three cancer types among uninsured adults (Fig 1). Asians had disproportionately lower rates of cervical and colorectal tests in 2008 and 2018. In addition, privately insured adults continued to have higher reported recent screening rates compared with uninsured, Medicare, and Medicaid and other public coverage groups (Fig 2). Compared with 2008, there is a significant improvement in reported colorectal cancer screening rates for all racial/ethnic and insurance groups in 2018.

### Regression analyses

#### Regression analysis for 2008

Table 3 presents the results of the logistic regressions examining the relationship between recent cancer screening by race/ethnicity and insurance in 2008 and 2018. The unadjusted Model 1 for 2008 indicated that racial/ethnic groups had lower odds of recent cervical tests and colorectal cancer screening than non-Hispanic Whites. Specifically, Hispanics and non-Hispanic Blacks had lower odds of receiving colorectal cancer screening, and Hispanics and non-Hispanic Asians had lower odds of receiving cervical tests in 2008 (Table 3). There were no statistically significant differences or disparities for Hispanics and non-Hispanic Asians in mammogram screening in 2008. However, non-Hispanic Blacks showed higher mammography rates than non-Hispanic Whites. In 2008, after adjusting for insurance status in Model 2, racial disparities in recent screening remained for cervical tests and colorectal screening. However, the disparity magnitude has attenuated in Model 2 compared with Model 1 for recent colorectal screening among Hispanics and non-Hispanic Blacks. Non-Hispanic Blacks no longer had disparities in colorectal cancer screening rates compared with non-Hispanic Whites after adjusting for insurance status in 2008. In addition, non-Hispanic Blacks have higher odds of recent mammography than non-Hispanic Whites. Uninsured and Medicare status were independently associated with lower odds of recent screening for all cancer tests compared with private insurance in 2008 (Table 3).

**Table 3:** Multiple logistic regressions: Associations between recent cancer screening, race/ethnicity, insurance status, and other factors–NHIS 2008 and 2018.

	Recent Cervical Screening			Recent Mammogram Screening			Recent Colorectal Screening		
	2008 OR (95% CI)	2018 OR (95% CI)	P-value†	2008 OR (95% CI)	2018 OR (95% CI)	P-value†	2008 OR (95% CI)	2018 OR (95% CI)	P-value†
**Model 1**									
**Race/Ethnicity**									
Black, N.H.	1.01 (0.79–1.29)	1.33 (1.02–1.74)*	0.139	1.27 (1.01–1.60)*	0.97 (0.76–1.24)	0.115	0.79 (0.67–0.94)**	0.82 (0.69–0.96)*	0.877
Hispanic	0.68 (0.55–0.84)***	0.87 (0.70–1.08)	0.111	0.77 (0.58–1.01)	0.96 (0.74–1.23)	0.236	0.43 (0.36–0.51)***	0.67 (0.56–0.80)***	0.000
Asian, N.H.	0.35 (0.26–0.48)***	0.54 (0.41–0.69)***	0.043	0.85 (0.53–1.35)	0.85 (0.57–1.26)	0.995	0.86 (0.63–1.17)	0.59 (0.46–0.78)***	0.076
White, N.H.	1.00	1.00		1.00	1.00		1.00	1.00	
**Model 2**									
**Race/Ethnicity**									
Black, N.H.	1.21 (0.94–1.57)	1.51 (1.14–2.00)**	0.251	1.45 (1.16–1.83)**	1.10 (0.85–1.41)	0.104	0.86 (0.72–1.02)	0.84 (0.71–1.00)	0.805
Hispanic	0.97 (0.78–1.22)	1.14 (0.91–1.44)	0.324	1.00 (0.76–1.32)	1.30 (0.98–1.72)	0.199	0.52 (0.43–0.62)***	0.77 (0.63–0.93)**	0.003
Asian, N.H.	0.33 (0.24–0.46)***	0.50 (0.39–0.65)***	0.043	0.86 (0.54–1.36)	0.86 (0.57–1.30)	0.997	0.87 (0.64–1.18)	0.59 (0.45–0.77)***	0.059
White, N.H.	1.00	1.00		1.00	1.00		1.00	1.00	
**Insurance**									
Uninsured	0.28 (0.24–0.34)***	0.29 (0.23–0.36)***	0.878	0.26 (0.20–0.35)***	0.17 (0.13–0.23)***	0.044	0.20 (0.15–0.27)***	0.22 (0.17–0.29)***	0.690
Medicaid/Other public	0.74 (0.57–0.97)*	0.79 (0.62–1.00)*	0.749	0.73 (0.49–1.07)	0.62 (0.47–0.81)***	0.503	0.82 (0.67–1.01)	0.93 (0.79–1.10)	0.348
Medicare	0.38 (0.26–0.57)***	0.35 (0.25–0.50)***	0.746	0.54 (0.43–0.67)***	0.64 (0.54–0.77)***	0.207	0.81 (0.70–0.94)**	1.18 (1.03–1.35)*	0.000
Private	1.00	1.00		1.00	1.00		1.00	1.00	
**Model 3**									
**Race/Ethnicity**									
Black, N.H.	1.48 (1.13–1.94)**	1.98 (1.47–2.68)***	0.162	1.77 (1.41–2.22)***	1.32 (1.02–1.71)*	0.152	1.02 (0.86–1.22)	1.04 (0.87–1.25)	0.854
Hispanic	0.96 (0.72–1.27)	1.25 (0.95–1.65)	0.141	1.18 (0.85–1.64)	1.38 (0.99–1.90)	0.126	0.72 (0.58–0.90)**	1.01 (0.79–1.28)	0.002
Asian, N.H.	0.28 (0.20–0.39)***	0.42 (0.32–0.55)***	0.050	0.72 (0.44–1.17)	0.75 (0.49–1.15)	0.837	0.80 (0.60–1.07)	0.55 (0.42–0.72)***	0.072
White, N.H.	1.00	1.00		1.00	1.00		1.00	1.00	
**Insurance**									
Uninsured	0.41 (0.34–0.50)***	0.43 (0.34–0.55)***	0.583	0.37 (0.27–0.51)***	0.22 (0.16–0.31)***	0.054	0.29 (0.22–0.40)***	0.32 (0.25–0.41)***	0.672
Medicaid/Other Public	1.34 (0.99–1.80)	1.35 (1.03–1.77)*	0.839	1.22 (0.80–1.84)	0.92 (0.69–1.22)	0.635	0.94 (0.75–1.17)	1.09 (0.91–1.30)	0.214
Medicare	0.93 (0.60–1.43)	0.84 (0.54–1.31)	0.813	1.00 (0.76–1.32)	0.86 (0.70–1.07)	0.446	0.78 (0.65–0.93)**	1.05 (0.89–1.24)	0.003
Private	1.00	1.00		1.00	1.00		1.00	1.00	
**Sex**									
Female	N/A	N/A		N/A	N/A		0.97 (0.85–1.10)	0.87 (0.79–0.97)*	
Male	N/A	N/A		N/A	N/A		1.00	1.00	
**Age group**									
21–44 years	1.21 (1.03–1.43)*	1.35 (1.15–1.59)***		N/A	N/A		N/A	N/A	
45–65 years	1.00	1.00		N/A	N/A		N/A	N/A	
50–64 years	N/A	N/A		0.93 (0.74–1.15)	0.87 (0.72–1.05)		0.58 (0.50–0.67)***	0.62 (0.54–0.70)***	
65–75 years	N/A	N/A		1.00	1.00		1.00	1.00	
**Education**									
Less than a high school diploma	1.06 (0.78–1.44)	0.90 (0.66–1.23)		0.88 (0.64–1.19)	0.99 (0.73–1.34)		0.72 (0.59–0.88)**	0.87 (0.72–1.06)	
Some college/ Associate degree	1.56 (1.26–1.94)***	1.31 (1.05–1.63)*		1.23 (0.99–1.53)	1.17 (0.96–1.42)		1.26 (1.07–1.48)**	1.17 (1.02–1.35)*	
Bachelor’s degree or higher	1.82 (1.40–2.37)***	1.75 (1.39–2.21)***		1.58 (1.20–2.08)**	1.70 (1.35–2.13)***		1.64 (1.38–1.95)***	1.46 (1.27–1.69)***	
High school diploma or GED	1.00	1.00		1.00	1.00		1.00	1.00	
**Marital Status**									
Not Married	0.67 (0.56–0.81)***	0.53 (0.43–0.64)***		0.76 (0.63–0.92)**	0.80 (0.66–0.97)*		0.89 (0.78–1.01)	0.69 (0.62–0.77)***	
Married	1.00	1.00		1.00	1.00		1.00	1.00	
**Employment**									
Not Employed	0.65 (0.53–0.80)***	0.60 (0.49–0.74)***		0.70 (0.57–0.87)**	0.88 (0.72–1.08)		1.18 (1.02–1.38)*	1.30 (1.16–1.47)***	
Employed	1.00	1.00		1.00	1.00		1.00	1.00	
**Family Income**									
≥$100,000	2.15 (1.54–2.99)***	1.48 (1.11–1.99)**		2.15 (1.52–3.03)***	1.55 (1.15–2.10)**		1.51 (1.22–1.88)***	1.37 (1.13–1.66)**	
$75,000-$99,999	1.59 (1.11–2.28)*	1.42 (1.03–1.97)*		1.53 (1.10–2.13)*	1.26 (0.93–1.70)		1.14 (0.91–1.43)	1.35 (1.12–1.64)**	
$35,000-$74,999	1.09 (0.89–1.34)	1.19 (0.95–1.50)		1.37 (1.10–1.70)**	1.23 (0.99–1.53)		1.24 (1.05–1.46)*	1.12 (0.97–1.31)	
$0 to $34,999	1.00	1.00		1.00	1.00		1.00	1.00	
**Health Status**									
Fair/Poor	0.95 (0.73–1.24)	1.13 (0.88–1.45)		0.86 (0.68–1.09)	0.81 (0.65–1.00)		1.20 (1.02–1.42)*	1.13 (0.98–1.31)	
Excellent/Very Good/Good	1.00	1.00		1.00	1.00		1.00	1.00	
**Language**									
Limited English proficiency	1.55 (1.04–2.30)*	1.35 (0.88–2.06)		1.05 (0.64–1.71)	1.33 (0.87–2.04)		0.68 (0.50–0.93)*	0.76 (0.55–1.06)	
English proficiency	1.00	1.00		1.00	1.00		1.00	1.00	

† P-value for the difference in effect size

*p ≤0.05

**p ≤0.01

***p≤0.001. Model 1 is a simple logistic regression model with race/ethnicity as the single predictor variable. Model 2 adds insurance status as a model adjustment factor. Model 3 additionally adjusts for age group, sex, education level, marital status, employment status, family income level, self-reported health status, and English language proficiency.

In 2008, after adjusting for other potential confounders in Model 3, racial/ethnic disparities in cancer screening remained in certain minority groups. Non-Hispanic Asians had lower odds of recent cervical tests (Odds Ratio [OR] = 0.28, 95% Confidence Interval [CI]: 0.20–0.39, p<0.001) than non-Hispanic Whites, and Hispanics had lower odds of recent colorectal cancer screening (OR = 0.72, 95% CI: 0.58–0.90, p<0.01), relative to non-Hispanic Whites. On the other hand, non-Hispanic Blacks had higher odds of recent cervical tests (OR = 1.48, 95% CI: 1.13–1.94, p<0.01) as well as recent mammogram tests (OR = 1.77, 95% CI: 1.41–2.22, p<0.001), relative to non-Hispanic Whites. In 2008, uninsured adults had significantly lower odds of cancer screening for all cancer types, and Medicare-insured adults had lower odds of colorectal cancer screening compared with privately insured adults (Table 3).

#### Regression analysis for 2018

In Table 3, the unadjusted Model 1 for 2018 indicated that non-Hispanic Blacks had higher odds of recent cervical tests, while non-Hispanic Asians had lower odds of recent cervical tests than non-Hispanic Whites. For colorectal cancer screening, non-Hispanic Blacks, non-Hispanic Asians, and Hispanics had lower odds of recent screening compared with non-Hispanic Whites in 2018. Racial/ethnic disparities were not observed for recent mammogram screening tests in 2018. In 2018, after adjusting for insurance status in Model 2, the racial/ethnic disparities in cancer screening remained for recent cervical and colorectal cancer tests. The magnitude of differences was partially reduced in Model 2 compared with Model 1 in 2018 (Table 3). Non-Hispanic Blacks no longer had disparities in colorectal cancer screening compared with non-Hispanic Whites after adjusting for insurance status. However, recent colorectal cancer screening disparities for non-Hispanic Asians and Hispanics persisted in 2018, along with cervical cancer screening disparities for non-Hispanic Asians. In 2018, Medicaid and Medicare insurance was also independently associated with lower odds of recent cervical test and mammogram screening compared with private insurance. Uninsured status was independently associated with lower odds for all cancer screening compared with private insurance in 2018 (Table 3).

In 2018, after adjusting for other potential confounders in Model 3, the odds ratio increased for certain race/ethnicity groups; non-Hispanic Blacks had higher odds of recent cervical tests and mammogram screening than non-Hispanic Whites. For all cancer screening, being Hispanic was not associated with significantly different odds for recent screening compared with non-Hispanic Whites in 2018. However, non-Hispanic Asians had lower odds of recent cervical tests (OR = 0.42, 95% CI: 0.32–0.55, p<0.001) and colorectal cancer screening (OR = 0.55, 95% CI: 0.42–0.72, p<0.001) relative to non-Hispanic Whites in 2018. On the other hand, non-Hispanic Blacks reported higher odds of cervical tests (OR = 1.98, 95% CI: 1.47–2.68, p<0.001) and mammogram screening (OR = 1.32, 95% CI: 1.02–1.71, p<0.05) than non-Hispanic Whites in 2018 (Table 3).

Significant differences in cancer screening by insurance status remained after adjusting for all other potential confounders. Specifically, Uninsured adults had lower odds of recent screening for all three cancers compared with privately insured adults in 2018. In 2018, Medicare-insured adults were not significantly different for all three-cancer screening compared with privately insured adults. Medicaid and other public coverage-insured adults had higher odds of receiving cervical tests and not-significantly different odds for a mammogram and colorectal cancer screening compared with privately insured adults in 2018 (Table 3).

#### Racial/Ethnic and insurance disparities in 2008 vs. 2018

As presented in Table 3, racial/ethnic disparities in colorectal cancer screening persisted in the unadjusted Model 1 analysis for 2008 and 2018. However, they were less pervasive for non-Hispanic Blacks and Hispanics between the two-time points. Non-Hispanic Asians reported a significant decrease in the odds of recent colorectal cancer screening in 2018, suggesting a magnification of disparities in colorectal cancer screening compared with non-Hispanic Whites between 2008 and 2018. Table 3 reports the results of the effect modification analysis, which indicate a statistically significant change in the effect sizes for Hispanics (p<0.001) and a marginally significant change for non-Hispanic Asians (p = 0.076) between 2008 and 2018. In the unadjusted Model 1, the Asian-White disparities in recent cervical tests and Hispanic-White disparities in recent colorectal screening were generally reduced or attenuated over this time. No significant changes in racial/ethnic group effect sizes for mammogram screening. After adjusting for other potential confounders in Model 3, the improvements in Hispanic-White disparities in colorectal cancer screening were no longer significant, suggesting that most of the progress was due to changes in insurance coverage and other sociodemographic characteristics of racial/ethnic groups. The Asian-White disparity in recent cervical test rates remained in 2018, despite statistically significant improvements in Asian-White disparities between 2008 and 2018 (p<0.05). In the fully adjusted Model 3, Hispanics had lower odds of recent colorectal cancer screening (OR: 0.72, 95% CI: 0.58–0.90, p<0.01) than non-Hispanic Whites in 2008, whereas Hispanics had comparable odds of recent colorectal cancer screening (OR: 1.01, 95% CI: 0.79–1.28, p = 0.96) in 2018, representing a significant change in relative proximity of recent odds ratios closer to ‘1’ (from 0.72 to 1.01) over time (p<0.01). In contrast, the result for non-Hispanic Asians suggests racial/ethnic disparities remain for recent cervical tests, and odds for colorectal cancer screening became statistically significant between 2008 and 2018. Specifically, non-Hispanic Asians had lower, but not statistically significantly different odds of recent colorectal cancer screening (OR: 0.80, 95% CI: 0.60–1.07, p = 0.137) than non-Hispanic Whites in 2008, whereas Asians had significantly lower odds of recent colorectal cancer screening (OR: 0.55, 95% CI: 0.42–0.72, p<0.001) in 2018, representing a decrease in effect sizes between the two time periods (Table 3). In 2008, non-Hispanic Asians had lower odds of recent cervical tests than non-Hispanic Whites (OR = 0.28, 95% CI: 0.20–0.39, p<0.001), whereas, in 2018, they still had lower odds of recent cervical screening (OR = 0.42, 95% CI: 0.32–0.55, p<0.001). These findings suggest that although changes in cancer screening rates attenuated over time (p<0.05), cervical cancer screening disparities among non-Hispanic Asians persisted in 2018. For non-Hispanic Blacks, the findings suggest no significant changes in the odds of screening for all three cancer types between 2008 and 2018. Insurance-based disparities between uninsured and privately insured adults did not appear to change over time for all cancer types. In 2008, Medicare-insured adults reported significantly lower odds of colorectal cancer screening compared with privately insured adults. In contrast, in 2018, Medicare-insured adults reported non-significant higher odds, representing a significant change in the effect size between 2008 and 2018 (p<0.001) (Table 3).

## Discussion

Results from this study indicate that rates of colorectal cancer screening have increased for all racial/ethnic groups and insurance groups between 2008 and 2018. However, the overall recent 2018 colorectal screening rate of 64.9% is lower than the Healthy People 2020 target of 70.5% [1]. Additionally, screening rates for cervical and mammogram tests between 2008 and 2018 have remained relatively stagnant for all racial/ethnic and insurance groups. In 2018, the overall recent mammogram and cervical screening rates were 73.0% and 80.0%, respectively, lower than the Healthy People 2020 target of 81.8% and 93% [1]. Our findings are further validated by the cancer screening rates reported by the Centers for Disease Control and Prevention (CDC) using NHIS data [69–71]. The observed decline in cervical screening rates in 2018 compared with 2008 can be partially attributed to a lack of access, lack of knowledge, and not receiving recommendations from healthcare professionals [72]. The present study offers a detailed analysis that reveals disparities and variations among race/ethnicity and insurance groups, which can inform targeted interventions and program development.

Disparities persist for specific race/ethnicity groups, even after adjusting for potential confounders in Model 3. Between 2008 and 2018, Non-Hispanic Asians experienced a significant change in the magnitude of disparities compared with Whites, as the odds ratio for cervical screening increased from 2008 (OR: 0.28, 95% CI: 0.20–0.39) to 2018 (OR:0.42, 95% CI: 0.32–0.55). Despite this improvement, non-Hispanic Asians continue to report lower odds of undergoing cervical compared with Whites over these two points. In 2008, non-Hispanic Asians reported non-significant lower odds of colorectal cancer screening compared with non-Hispanic Whites (OR: 0.80, 95% CI: 0.60–1.07, p = 0.137), although by 2018, the odds significantly decreased (OR: 0.55, 95% CI: 0.42–0.72, p<0.001), indicating a widening disparity between the two groups. This study’s results are consistent with other publications that found lower rates of cervical and colorectal tests in non-Hispanic Asians compared with other racial/ethnic groups using large-scale public health survey data over time [15, 19, 73–76]. For example, a recent Health Information National Trends Survey (HINTS) paper examining data from 2011–2014 reported that Asians had lower odds of cervical cancer screening compared with Whites (OR: 0.66, 95% CI: 0.66–0.67) [74]. In contrast to our findings which reported significantly lower odds of cervical screening, a Behavioral Risk Factor Surveillance System (BRFSS) study assessing 2015 and 2016 found a non-significant association between Asians and cervical cancer screening compared to whites (OR for no recent screening: 1.27 [95% CI: 0.94–1.70]), although the direction of the disparity is aligned with our findings. The observed differences compared to our findings may be attributed to an underrepresentation of Asians (<3% of study population). Oversampling to boost the representation of the Asian population may allow the study to have sufficient power to detect significant differences. The BRFSS’s model adjustment was also limited to income, education, and rurality. It did not consider other factors associated with lower odds of cervical cancer screening among Asians, such as older age, poverty status, uninsured status, unmarried, no usual source of care, lower Asian American community density, farther distance to screening, and lower supply of providers [77–84].

Historically, non-Hispanic Blacks have reported lower colorectal cancer screening rates compared with non-Hispanic Whites; however, considerable evidence suggests these disparities have been attenuating over time [85–87]. Some studies have reported odds of colorectal screening among blacks that are comparable to or higher than non-Hispanic Whites, which aligns with our adjusted findings [45, 88–90]. Our findings reported that non-Hispanic Blacks have higher odds of cervical tests and mammograms than non-Hispanic Whites. These findings are consistent with the published literature, which has reported higher cervical and breast cancer screening rates among Black women for many years [72, 89–93]. For example, one recent NHIS study assessing 13 years between 2005–2018 found that Blacks had significantly higher odds of recent pap test (OR: 1.83, 95% CI: 1.69–1.99) and recent mammogram (OR: 1.47, 95% CI: 1.36–1.59) compared to Whites [94], consistent with our findings. However, a 2018 BRFSS study reported consistently higher cancer screening rates than those observed in our study for non-Hispanic Blacks, with breast cancer screening at 83.9% compared to ours at 72.2% and cervical cancer screening at 84.8% compared to ours at 82.5%. The observed differences may be attributed to limitations inherent in state-level BRFSS databases in general, which have been shown to overestimate the prevalence of cancer screening compared to the national-level NHIS databases [87, 90]. The aggregation of state-level BRFSS estimates to generate national estimates may introduce bias, potentially accounting for the higher rates of cervical and breast screening rates observed in the BRFSS study [87, 90].

Between 2008 to 2018, there is evidence to suggest that the disparity in colorectal cancer screening between Hispanic and White individuals has attenuated (p<0.01), with Hispanic individuals now having comparable odds of receiving screening with non-Hispanic White individuals (Table 3). These results align with the trends observed over time in the NHIS database [95] and the BRFSS database [90, 96], as reported in recent publications. For instance, a recent NHIS study reported that colorectal screening rates increased from approximately 47% in 2010 to 57.6% in 2018 [95], consistent with our findings which reported 36.7% in 2008 to 59.5% in 2018.

Having established that racial/ethnic disparity have attenuated for certain minority groups, it is worth examining the role of government funding and insurance coverage on cancer screening rates. National-level government-funded programs such as the National Breast and Cervical Cancer Early Detection Program (NBCCEDP) [97] and the Colorectal Cancer Control Program (CRCCP) [98] improve the uptake of screening in the low-income, uninsured, and underinsured populations and may account for increased rates among non-Hispanic Blacks and other racial/ethnic minorities [11, 89, 90, 99, 100]. In addition, the passage of the Patient Protection and Affordable Care Act (ACA) of 2010 and the subsequent reduction in the cost of care and improved access to screening services have helped minority populations with the cervical, mammogram, and colorectal screening uptake [86, 101–104]. For instance, a systemic review of ACA’s impact on colorectal cancer screening behaviors found that non-Hispanic Blacks and Hispanics experienced greater increases in colorectal cancer screening compared with Whites after the passage of the ACA [104]. The ACA played an essential role in increasing cancer screening rates by expanding Medicaid eligibility to encompass millions of low-income individuals who were previously uninsured and disproportionately from race/ethnicity minority groups. Furthermore, the ACA requires health plans to include coverage for cancer screening and other preventive services and eliminate cost-sharing requirements for beneficiaries. One NHIS study found that ACA’s removal of financial barriers increased colorectal cancer screening prevalence between 2008 and 2013 among low-income least-educated individuals [105]. This suggests that the elimination of cost-sharing provisions may have reduced costs and increased accessibility, which in turn has contributed to the reduction of race/ethnicity disparities in cancer screening. However, the extent of disparities reduction may be influenced by the varying implementation of Medicaid expansion at the state level.

The ACA is also important in addressing disparities in cancer screening rates between insured and uninsured individuals. Between 2008 and 2018, our study found persistent disparities in cancer screening rates between uninsured and privately insured adults, with significant differences in screening rates observed across all three cancer types. Compared with privately insured adults, Medicare and Medicaid and other public coverage adults reported lower rates of cervical and mammogram screenings while having similar rates of colorectal screening in 2018. These findings are consistent with previously published results [106, 107], such as CDC analysis that used the NHIS database, which found similar disparities in screening rates between private, public, and uninsured adults [69–71]. Furthermore, our study found that after adjustment for potential confounding factors in Model 3, there was no significant difference in the odds of cancer screening between Medicare and Medicaid and other public coverage compared with privately-insured adults in 2018.

The Medicaid continuous coverage provision under the Families First Coronavirus Response Act of 2020 (FFCRA) provides enhanced federal funding to states during the coronavirus disease 2019 (COVID-19) public health emergency (PHE). Renewed attention on Medicaid coverage gaps is necessary and inevitable as the FFCRA is set to expire, particularly in U.S. states that have not already expanded Medicaid eligibility under the ACA. Experts have estimated that as many as 15 million Medicaid beneficiaries may lose their healthcare coverage when FFCRA’s funding provisions expire, and the COVID-19 PHE ends [108–110]. States that have no expanded Medicaid should consider the potential benefits of adopting Medicaid expansion under the ACA to offset the coverage loss caused by the end of FFRCA funding. Medicaid expansion would extend healthcare coverage to millions of low-income individuals and reduce the number of uninsured, which could improve cancer screening uptake and reduce screening disparities [111, 112]. In 2019, approximately 60% of the Medicaid coverage gap population were racial/ethnic minorities, reflecting enduring disparities in healthcare access that expanded coverage would do much to address [113].

Our study’s results highlight persistent racial/ethnic disparities in cancer screening between non-Hispanic Asians and non-Hispanic Whites, with non-Hispanic Asians consistently reporting lower cervical and colorectal screening rates. The findings reinforce the need for targeted interventions toward eliminating disparities and improving cancer screening among underserved groups, such as non-Hispanic Asians. Minority groups, especially those uninsured, may benefit from targeted intervention aimed at increasing insurance coverage to increase cancer screening uptake. As previously addressed, the ACA’s expansion of Medicaid, for instance, would provide medical coverage to a considerable number of disadvantaged persons and those without insurance. Furthermore, the cause of underutilization of cervical tests among Asians may be due to cultural attitudes and beliefs, language barriers, lack of routine healthcare access, or lack of knowledge of cervical cancer and preventative services [11, 19]. Designing culturally-sensitive interventions that target these barriers may improve cervical test rates among Asians. Potential strategies to enhance screening rates in non-Hispanic Asians could include community-based or workplace-based group educational programs, enhancing cultural awareness among healthcare professionals, and partnering with outreach workers to overcome language and cultural barriers [114, 115]. One cluster-randomized controlled trial demonstrated that community health worker-led health-literacy intervention, involving personalized health-literacy training, cancer-screening brochures, and monthly telephone counseling, significantly increased cervical (OR: 13.3, 95% CI: 7.5–22.3) screening uptake and positive perceptions about cancer screening among Korean-American women [116]. Through the implementation of these and other similar interventions, healthcare professionals can work together to reduce racial/ethnic disparities in cervical and colorectal cancer screening among non-Hispanic Asians and ultimately promote health equity.

The present study has several limitations. The results are a cross-sectional snapshot in time, so longitudinal follow-up to assess the patient-level preventive service utilization over time was not possible. Moreover, estimating national levels of cancer screening prevalence limits inferences about individual risks and behaviors. Additionally, although the analysis accounted for various potential confounding factors, other covariates were unavailable due to the secondary observational nature of the dataset (e.g., barriers, knowledge, or beliefs about cancer screening, risk factor profiles, and patient-provider communication on cancer screening). These factors may play an essential role in explaining some of the remaining racial/ethnic or insurance disparities in the present analysis [19, 117]. The final response rate among adults was 62.6% and 53.1% in 2008 and 2018, respectively, and non-response bias may exist despite survey weight adjustments [56]. Due to how the questions were asked, we cannot distinguish between a screening and a diagnostic exam for each cancer type. Survey questions about tests and USPSTF recommendations for using them have changed and evolved over time, reflecting trends in evidence-based practice and technology. Finally, recall bias is a limitation of any retrospective study relying on self-reported answers from survey respondents. Despite these limitations, the present study leverages a nationally representative public health survey and a large sample size for various racial/ethnic minority groups to document enduring disparities in preventive care utilization based on race/ethnicity and insurance status.

## Conclusion

Overall, the study results demonstrate that despite the increase in breast and colorectal cancer screening rates between 2008 and 2018, persistent racial/ethnic disparities exist among race/ethnicity and insurance groups. Non-Hispanic Asians consistently reported lower odds of cervical and colorectal screening compared with non-Hispanic Whites between 2008 and 2018, indicating that targeted intervention is needed in this underserved group. On the other hand, Non-Hispanic Blacks reported higher odds of recent cervical and mammograms than non-Hispanic Whites, which is consistent with previously published literature. While improvements in colorectal cancer screening were observed among Hispanics over time, no significant changes in odds of screening were observed for cervical and breast cancer among this population. Uninsured status was associated with significantly lower odds of cancer screening across the two time periods for all three types of cancers. These findings highlight the importance of understanding and addressing the underlying factors contributing to disparities in cancer screening utilization among race/ethnicity and insurance groups. Targeted intervention programs such as community-based group educational programs and culturally sensitive outreach efforts may reduce disparities and enhance cancer screening uptake rates. Government-funded initiatives such as Medicaid expansion under the ACA could increase access to healthcare for millions of low-income and uninsured individuals and may eventually help reduce disparities. Addressing the disparities in cancer screening rates among the underserved population is essential to reducing the disease burden of cancer, promoting health equity, and improving overall cancer outcomes in the U.S.

## Supporting information

S1 Checklist STROBE statement—checklist of items that should be included in reports of observational studies. (DOCX)
