# Supplementary material for: Examining cancer screening disparities by race/ethnicity and insurance groups: A comparison of 2008 and 2018 National Health Interview Survey (NHIS) data in the United States
Source: PLoS One. 2024 Feb 28;19(2):e0290105. doi: 10.1371/journal.pone.0290105 (PMC10901319; doi:10.1371/journal.pone.0290105)
Supplement: S1 Checklist — (DOCX) [file pone.0290105.s001.docx]

**STROBE Statement—checklist of items that should be included in reports of observational studies.**

**Study Title:** Examining cancer screening disparities by race/ethnicity and insurance groups: A comparison of 2008 and 2018 National Health Interview Survey (NHIS) data in the United States

**Study Design:** Cross-sectional study

|  | Item No. | Recommendation | Addressed (Y/N), Page  No. | Relevant text from manuscript |
| --- | --- | --- | --- | --- |
| **Title and abstract** | 1 | (*a*) Indicate the study’s design with a commonly used term in the title or the abstract | Yes, page 2 | The study design is indicated in the methods section of the abstract: “*A retrospective cross-sectional analysis of the 2008 and 2018 National Health Interview Survey (NHIS) database…”* Page 2, line 40-41 |
|  |  | (*b*) Provide in the abstract an informative and balanced summary of what was done and what was found | Yes, page 2-3 | This information is stated in study abstract as methods, results and conclusion described. Page 2-3, line 35-64 |
| Introduction | | | |  |
| Background/rationale | 2 | Explain the scientific background and rationale for the investigation being reported | Yes, page 3-4 | Rationale and existing literature are stated in the introduction section. Page 3-4, line 66-99. |
| Objectives | 3 | State specific objectives, including any prespecified hypotheses | Yes, page 5 | This information is specified in the objectives section:  “*The study's objective is to assess the relationship between cancer screening rates, race/ethnicity, and insurance coverage and to quantify the changes in screening disparities in 2008 compared with 2018 for breast, cervical, and colorectal cancer using the National Health Interview Survey (NHIS) database*.” Page 5, line 101-104 |
| Methods | | | |  |
| Study design | 4 | Present key elements of study design early in the paper | Yes, page 5 | The study design is stated in the first subsection of methods. All key elements are described in the methods section.  *“This study is a retrospective cross-sectional analysis of data from the 2008 and 2018 National Health Interview Survey (NHIS)”* Page 5, line 107-108 |
| Setting | 5 | Describe the setting, locations, and relevant dates, including periods of recruitment, exposure, follow-up, and data collection | Yes, page 5 | Setting, location, time-period, data source, and data collection are fully described in the Data Sources subsection on page 5.   - Setting, location, time-period, and data collection: *“The NHIS is a cross-sectional household interview survey providing health information on the civilian noninstitutionalized population in the U.S. The National Center for Health Statistics (NCHS) of the Centers for Disease Control and Prevention (CDC) collects the data annually.”* Page 5, line 108-111. - Time-period: *“This study is a retrospective cross-sectional analysis of data from the 2008 and 2018 National Health Interview Survey (NHIS)”* Page 5, line 107-108 |
| Participants | 6 | (*a*) *Cohort study*—Give the eligibility criteria, and the sources and methods of selection of participants. Describe methods of follow-up  *Case-control study*—Give the eligibility criteria, and the sources and methods of case ascertainment and control selection. Give the rationale for the choice of cases and controls  *Cross-sectional study*—Give the eligibility criteria, and the sources and methods of selection of participants | Yes, page 6 | The present study is a cross-sectional study. The study population is described in the methods section (Data sources subsection).  “*All adults who responded to the cancer control supplement of the NHIS and answered questions related to breast, cervical, and colorectal cancer screening will be included in the study.”* Page 6, 124-126. |
|  |  | (*b*) *Cohort study*—For matched studies, give matching criteria and number of exposed and unexposed  *Case-control study*—For matched studies, give matching criteria and the number of controls per case | Not applicable | Not applicable; no matching involved. |
| Variables | 7 | Clearly define all outcomes, exposures, predictors, potential confounders, and effect modifiers. Give diagnostic criteria, if applicable | Yes, page 6-8 | Variable definitions for outcomes and predictors were fully described in the Measures section, specifically in the Primary outcomes subsection (page 6), Primary predictors subsection (page 7), and Secondary predictors subsection (page 8).  Examples:   - *“The primary outcome is self-reported preventive care utilization which three cancer screening indicators will measure……. Each outcome will be dichotomized into "recent screening" versus "no recent screening."”* Page 6, line 131-136. - *“The main independent variables will be race/ethnicity and types of health insurance coverage. Race/ethnicity will be categorized into Hispanic, Non-Hispanic White, Non-Hispanic Black, and Non-Hispanic Asian, as reported by respondents.”* Page 7, line 152-154. |
| Data sources/ measurement | 8* | For each variable of interest, give sources of data and details of methods of assessment (measurement). Describe comparability of assessment methods if there is more than one group | Yes, page 5-8 | All outcomes and predictors were based on interview answers to survey questions from each respondent, as described in the Methods section.   - *“The survey consists of a core set of interview questions that gathers baseline demographics, socioeconomic, and health status characteristics for each member of the household.”* Page 5, line 112-114. - *“During the interview, respondents were asked, "When did you have your most recent [screening test]?" and those who responded affirmatively were also asked the month and the year of the recent screening test.”* Page 6, line 133-135. |
| Bias | 9 | Describe any efforts to address potential sources of bias | Yes, page 9-11 | The study notably tried to address sources of bias by using statistical adjustment for confounding variables. *“Other potential confounders were adjusted in Model 3, including sex, age, education level, marital status, employment status, family income level, self-reported health status, and English language proficiency. Model 3 allowed us to assess the effect of potential confounders on the relationship between race/ethnicity, insurance types, and cancer screening utilization”* Page 10, line 221-225. |
| Study size | 10 | Explain how the study size was arrived at | Yes, page 6 | The methods describe the inclusion of two NHIS database years. The sample is further described in Table 2.  *“In 2008 and 2018, the total unweighted sample sizes were 21,781 and 25,417 adults 18 and older, respectively.”* Page 6, line 121-122. |
| Quantitative variables | 11 | Explain how quantitative variables were handled in the analyses. If applicable, describe which groupings were chosen and why | Yes, page 8-9 | As described in the methods section, variables were defined and grouped based on previously published literature.  *“Individuals with more than one type of health insurance were assigned to the first appropriate hierarchy as previously described. [50, 51, 62]”* Page 8, line 167-169.  *“The grouping categories chosen are in line with previously conducted research.* *[11, 38, 45, 53]”* Page 9, line 185-186. |
| Statistical methods | 12 | (*a*) Describe all statistical methods, including those used to control for confounding | Yes, page 8-11 | These are described in the Methods section, specifically the Statistical analysis subsection.  *“A three-step logistic regression model was conducted to assess the association between recent cancer screening, race/ethnicity, and types of insurance by analyzing 2008 and 2018 NHIS data separately. In Model 1, a simple logistic regression was performed to assess the total impact of race/ethnicity (main independent variable) on cancer screening. In Model 2, logistic regression was performed to assess the combined impact of race/ethnicity and insurance types (two main independent variables) on cancer screening…. Other potential confounders were adjusted in Model 3, including sex, age, education level, marital status, employment status, family income level, self-reported health status, and English language proficiency.”* Page 9-10, line 209-223.  *“A bivariate analysis Rao-Scott Chi-square test was performed to compare the distribution of cancer screening rates across race/ethnicity and insurance groups for 2008 and 2018 separately. A similar chi-square test was conducted to compare age-adjusted cancer screening rates in 2008 and 2018 among race/ethnicity and insurance groups.”* Page 9-10, line 202-204. |
|  |  | (*b*) Describe any methods used to examine subgroups and interactions | Yes, page 10-11 | This is described in the Methods section, specifically under the Statistical analysis subsection.  *“…Specifically, 2008 and 2018 data were combined for each cancer screening type. Dummy variables for each racial/ethnic group, insurance group, and two years were added as main effects. Each race/ethnicity X year and insurance group X year interaction terms were added to the model.”* Page 11, line 227-230 |
|  |  | (*c*) Explain how missing data were addressed | Yes, page 9 | This is described in the Methods section, specifically under the Statistical analysis subsection.  *“Responses initially classified as “Refused”, “Not Ascertained”, or “Don’t Know” for any cancer screening-related questions were re-coded as a “Missing” value. To account for missing values, the NOMCAR option was used in SAS survey procedures to treat missing values as not missing completely at random (NOMCAR). When the NOMCAR option and the DOMAIN statement are specified, the procedure computes variance estimates by analyzing the non-missing values as a domain or subpopulation of interest, accounting for missing and non-missing values in the overall population. As a result, no observations were dropped from the dataset to ensure proper calculation of variance estimates.”* Page 9, line 192-200. |
|  |  | (*d*) *Cohort study*—If applicable, explain how loss to follow-up was addressed  *Case-control study*—If applicable, explain how matching of cases and controls was addressed  *Cross-sectional study*—If applicable, describe analytical methods taking account of sampling strategy | Yes, page 9-10 | This study is a cross-sectional study. This is described in the Statistical analysis subsection.  *“The statistical analyses were performed using SAS 9.4 (SAS Institute, Cary, NC) and SAS survey procedures, specifically PROC SURVEYMEANS, SURVEYFREQ, SURVEYREG and SURVEYLOGISTICS. These SAS survey procedures were employed to properly account for the complex sampling design.”* Page 9, line 188-191. |
|  |  | (*e*) Describe any sensitivity analyses | Not applicable | Not applicable |
| Results | | | | |
| Participants | 13* | (a) Report numbers of individuals at each stage of study—eg numbers potentially eligible, examined for eligibility, confirmed eligible, included in the study, completing follow-up, and analysed | Yes, page 11 | This is described at the beginning of the results section.  *“In 2008, a weighted total of 76,769,989 adults aged 50-75 were eligible for colorectal cancer screening and included in the analysis compared with 95,778,802 in 2018. The weighted number of females aged 21-65 eligible for cervical screening increased from 73,390,857 in 2008 to 81,794,923 in 2018, and the weighted number of females aged 50-74 eligible for mammogram increased from 37,003,082 in 2008 to 46,130,514 in 2018.”* Page 11, line 234-239. |
|  |  | (b) Give reasons for non-participation at each stage | Yes, page 11 | See above, described in the results section. |
|  |  | (c) Consider use of a flow diagram | Not applicable | Use of a flow diagram was not deemed necessary given the simplicity of the database. |
| Descriptive data | 14* | (a) Give characteristics of study participants (eg demographic, clinical, social) and information on exposures and potential confounders | Yes, page 11-12 | Table 1 describes the baseline demographic and characteristics of study participants. |
|  |  | (b) Indicate number of participants with missing data for each variable of interest | Yes, page 11-12 | The total numbers of recorded data for each variable are stated in variable headline of each table |
|  |  | (c) *Cohort study*—Summarise follow-up time (eg, average and total amount) | Not applicable | Not applicable; This is a cross-sectional study |
| Outcome data | 15* | *Cohort study*—Report numbers of outcome events or summary measures over time | Not applicable | Not Applicable |
|  |  | *Case-control study—*Report numbers in each exposure category, or summary measures of exposure | Not applicable | Not Applicable |
|  |  | *Cross-sectional study—*Report numbers of outcome events or summary measures | Yes, page 11-12,14,17-19 | All numbers are reported in Table 1, 2 and 3. |
| Main results | 16 | (*a*) Give unadjusted estimates and, if applicable, confounder-adjusted estimates and their precision (eg, 95% confidence interval). Make clear which confounders were adjusted for and why they were included | Yes, page 17-19 | All adjusted odds ratio estimates, and 95% confidence intervals, are reported in Table 3. Non-adjusted odds ratio estimates are also displayed in Model 1 of Table 3, and Non-adjusted rate estimates (age-standardized) are displayed in Table 2. The caption at the end of Table 3 describes which confounders were adjusted in Models 1, 2, and 3. |
|  |  | (*b*) Report category boundaries when continuous variables were categorized | Yes, page 11-12, 18. | Category boundaries are displayed in the variable heading in the Table 1 and 3. The only continuous variable that was categorize was age groups. |
|  |  | (*c*) If relevant, consider translating estimates of relative risk into absolute risk for a meaningful time period | N/A | Not applicable; Odds Ratios were used, not relative risk. |
| Other analyses | 17 | Report other analyses done—eg analyses of subgroups and interactions, and sensitivity analyses | Yes, page 17-19 | This information is reported in Table 3. |
| Discussion | | | | |
| Key results | 18 | Summarise key results with reference to study objectives | Yes, page 23-30 | Key results are summarized in the Discussion section (page 23-30). The key findings are further summarized in the Conclusion section (page 30-31). The implication of study results on study objectives are noted in the discussion section.  *“The present study offers a detailed analysis that reveals disparities and variations among race/ethnicity and insurance groups, which can inform targeted interventions and program development.”* Page 24, line 431-433. |
| Limitations | 19 | Discuss limitations of the study, taking into account sources of potential bias or imprecision. Discuss both direction and magnitude of any potential bias | Yes, page 29-30 | Descriptions of the limitations is described in the Discussion section.  *“The present study has several limitations. The results are a cross-sectional snapshot in time, so longitudinal follow-up to assess the patient-level preventive service utilization over time was not possible….”* Page 29-30 line 559-576. |
| Interpretation | 20 | Give a cautious overall interpretation of results considering objectives, limitations, multiplicity of analyses, results from similar studies, and other relevant evidence | Yes, page 23-30 | The discussion section included references to previously published literature. Discrepancies with other studies was discussed in detail. Limitations were taken into account in the discussion.  *“This study's results are consistent with other publications that found lower rates of cervical and colorectal tests in non-Hispanic Asians compared with other racial/ethnic groups using large-scale public health survey data over time.[15, 19, 74-77]”* Page 24, line 443-445 |
| Generalisability | 21 | Discuss the generalisability (external validity) of the study results | Yes, page 30 | Study results are generalizable at the U.S. national level given the large sample size and complex, multistage, probability sample design of the NHIS database.  *“Despite these limitations, the present study leverages a nationally representative public health survey and a large sample size for various racial/ethnic minority groups to document enduring disparities in preventive care utilization based on race/ethnicity and insurance status.”* Page 30, line 573-576. |
| Other information | |  | | |
| Funding | 22 | Give the source of funding and the role of the funders for the present study and, if applicable, for the original study on which the present article is based | Yes, via submission system | Funding disclosure was provided at the time of submission, but not included in the manuscript in accordance with PLOS ONE guidelines. There was no funding was received for this research. |

*Give information separately for cases and controls in case-control studies and, if applicable, for exposed and unexposed groups in cohort and cross-sectional studies.

**Note:** An Explanation and Elaboration article discusses each checklist item and gives methodological background and published examples of transparent reporting. The STROBE checklist is best used in conjunction with this article (freely available on the Web sites of PLoS Medicine at http://www.plosmedicine.org/, Annals of Internal Medicine at http://www.annals.org/, and Epidemiology at http://www.epidem.com/). Information on the STROBE Initiative is available at www.strobe-statement.org.
